# COVID-19 and Pretentious Psychological Well-Being of Students: A Threat to Educational Sustainability

**DOI:** 10.3389/fpsyg.2020.628003

**Published:** 2021-01-27

**Authors:** Hui Li, Hira Hafeez, Muhammad Asif Zaheer

**Affiliations:** ^1^Business School, Hohai University, Nanjing, China; ^2^Department of Management Sciences, COMSATS University Islamabad, Lahore Campus, Lahore, Pakistan; ^3^Institute of Management Sciences, PMAS-Arid Agriculture University Rawalpindi, Rawalpindi, Pakistan

**Keywords:** COVID-19, economic sustainability, university students, psychological wellbeing, mental health, physical symptoms, home quarantine activities, COVID-19 stressors

## Abstract

Since the outbreak of COVID-19, reaction quarantine, social distancing, and economic crises have posed a greater risk to physical and psychological health. Such derogatory mental health stigma is associated with adverse outcomes in the student population. The purpose of the current study is to provide a timely evaluation of the COVID-19 pandemic and its adverse effects on students’ psychological well-being to sustain economic sustainability. A thorough review of the literature and current studies, significant emphasis of socio-demographic indicators, interpretation of physical symptoms, home quarantine activities, and COVID-19 unique stressors were extracted. Data were collected through electronic surveys from 640 university students at local and foreign universities. The findings revealed substantial adverse effects resulting in varying levels of stress, symptoms of depression, and specific discomfort in the case. Among COVID-19 stressors, financial instability, unpredictability toward future/career, and media exposure have been described as common factors that cause poor psychological well-being and weaken economic sustainability. COVID-19, quarantine, self-isolation, and onerous interventions primarily weaken university students’ mental health. The emphasis on this vulnerable category, however, is substantially absent from the literature. This research addresses the urgent need to develop possible solutions and preventive measures to promote economic sustainability by ensuring students’ psychological well-being.

## Introduction

In recent months, there have been many drastic changes that have changed how we live and how we die (Nyatanga, 2020; Wang et al., 2020). Cautions are becoming a part of life linked to the present COVID-19 pandemic situation (Nelson et al., 2020). Social media has made it difficult to stay away from reality and participate in debates (Shaw, 2020). These ongoing highlights and media debates make it difficult for each person to think about something else (Hossain et al., 2020). There is no question that the consequences of the present pandemic are not limited to physical limits but also psychological and mental well-being (Duan and Zhu, 2020). During this period, it is normal for the general population to enter a state of acute stress and depression (While and Nightingale, 2020). Normal reactions to some unnatural situations result from a high degree of stress and anxiety (Roy et al., 2020). The underlying reasons for initiating poor psychological well-being and disturbed mental health are an escalation of infected cases, high mortality rate, and massive quarantine (Bao et al., 2020; Rubin and Wessely, 2020).

In line with the proposed agenda, recent research highlighted that quarantine people might experience psychological distress (Brooks et al., 2020). In the context of post-traumatic stress symptoms, this condition of confusion and volatility has induced psychological distress in university students (Cao et al., 2020). It is hardly unlikely for a college/university student to escape physically and mentally from the detrimental consequences of the COVID-19 pandemic. For university students, psychological stress is increased primarily because of performance pressure, potential uncertainty, and academic disruption (Zhai and Du, 2020a). With every second, its insidious influence affects their psychological and mental well-being in the form of elevated stress levels, anxiety, symptoms of depression, and some event-specific distress (Bao et al., 2020). However, to meet the current requirements, educational arrangements are being created (Khan et al., 2020). However, in the present pandemic scenario, traditional norms and related performance requirements are becoming increasingly difficult to match. University approach for handling students and steering their emotions during general well-being emergencies and keeping away from misfortunes triggered by emergencies has become an urgent concern (Cao et al., 2020). There is an immediate need to consider the implications of this ongoing global pandemic on the student community’s mental health. In addition to this, there has been an emergence of research addressing pandemic impacts on healthcare workers or the general population in particular (Chen et al., 2020; Li et al., 2020b; Yang et al., 2020). There is, however, a shortage of such research focusing directly on the mental well-being of students during a pandemic situation (Son et al., 2020).

A recent study highlighted that during quarantine, and even after that period, students decided on poor psychological wellbeing and mental illness (Bao et al., 2020). A number of students have discussed about their experiences of stress and anxiety which they mainly facing because of academic work delays and lack of socialization (Cao et al., 2020). The propensity for stress or depression symptoms worsens due to radical changes in economic stability, especially in the job market (Anjum et al., 2019). In comparison, students have referred to agitation as post-isolation symptoms (Brooks et al., 2020). Approximately 83% of respondents agreed regarding their poor psychological well-being during quarantine (YoungMinds, 2020), according to YoungMind’s recent findings taken *via* a worldwide survey. Besides, a recent study presented troubling figures that one in four university students had poor mental well-being and psychological well-being (Odriozola-González et al., 2020).

The effect of COVID-19 causes an academic disturbance, low motivation, and high pressure on university students while concentrating on maintaining their academic performance (Khan et al., 2020; Son et al., 2020). Educational institutions are still ineffective in predicting the effects of pandemic stressors on coping strategies and learning abilities for learners. Simply stated, the COVID-19 pandemic has positioned students with an unparalleled mental health burden, urgently requiring further review and immediate intervention. However, the literature lacks the technique to learn about certain psychological disorders and their negative consequences during the pandemic (Brooks et al., 2020). Therefore, a timely call to action is proposed that how the COVID-19 pandemic is disturbing student mental health in terms of their academic results, career plans, and future growth (Khan et al., 2020). Also, how stress, anxiety, depression, and event-specific distress (ESD) as a subscale of psychological well-being and mental health are mainly getting triggered in the presence of observed predictors (Hossain et al., 2020). Narcissism positively influences unethical pro-organizational behavior (Shah et al., 2020). Workplace ostracism has a positive relationship with stress (Sarfraz et al., 2019b). Organizational justice plays a partial mediating role in the employee’s perception of CSR and employee’s outcomes (Sarfraz et al., 2018).

Furthermore, it is a fact that countries with low and medium incomes, such as Pakistan, have insufficient resources to tackle both COVID-19 and the numerous mental health problems arising from the outbreak. Barley is based on this overlooked population in these South Asian countries. The current study’s additional objective is to reduce this gap by providing comprehensive data, especially related to students from developed countries and their psychological health. Laterally, as suggested by Son and his colleagues (Son et al., 2020), it reacts to the disparities in mental health problems with sociodemographic features. This report also includes a substantial investigation into the ineffective treatment of students’ psychological and mental health concerns. The emphasis is on understanding certain troubling factors and inadequate implementations of customary coping mechanisms that delay academic institutions’ adverse academic and psychological results. As COVID-19 specific literature have consistent support and call for action in this adverse situation (Li et al., 2020a; Zhai and Du, 2020b).

## Methodology

### Research Design and Study Participants

A cross-sectional approach was implemented to develop the prerequisites for information related to the COVID-19 pandemic and its impact on university students’ psychological well-being and mental health. For data collection to meet the maximum number of participants in a limited period, the snowball sampling approach was adopted. First of all, these respondents were contacted by email and asked to provide their real experience of COVID-19 and its effect on their psychological and mental well-being (Li et al., 2020b). Later, the research team asked respondents *via* social media accounts or Whatsapp groups for their voluntary contribution to the distribution of questionnaires. It may take longer than face to face (Zhang and Ma, 2020) to approach students *via* electronic media. However, the measures were adopted to prevent virus infection and health risk that can be developed through physical contact.

**Figure 1:**
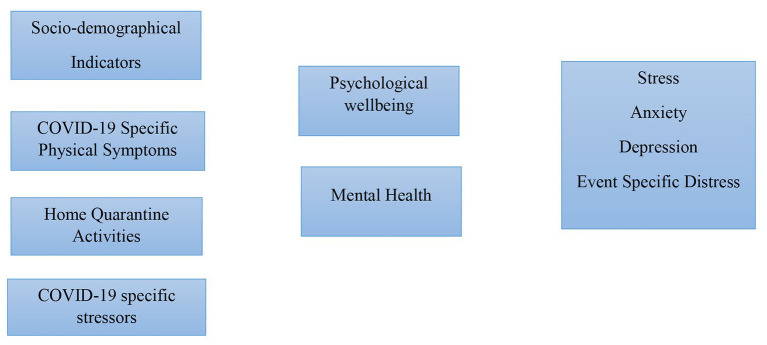
Covid-19 pandemic and university student study model.

The Figure 1 is depicting such factors (SDI, COVID-19 SPS, HQA, and COVID-19 SS) which are potentially effecting the psychological wellbeing and mental health of students in particular. It also highlighting the main concerns of student’s mental health in the form of stress, anxiety, depression, and event specific distress.The data collection process began on May 2, 2020, and ended on May 16, 2020. Participants were chosen from either local or foreign university after consenting to the eligibility requirements of being home quarantined and currently pursuing a degree. It was entirely voluntary, and without offering any reason, the participant could withdraw from the survey at any time. To begin with, participants were told that these surveys had no financial or non-financial benefits. In addition, their identity is kept secret and will not be used on other websites or for any other reason. Before starting the investigation, respondents gave their informed consent after going through the study’s objectives and intent and completed a self-reporting questionnaire. Six hundred forty responses from a total of 678 responses were accepted for final review after initial screening. This study was performed online in complete accordance with the Helsinki Declaration concerning human participant testing. The study involving human participants were reviewed and approved by the COMSATS University Islamabad. The participants provided their written, informed consent to participate in this study.

### Research Measures

#### Sociodemographic Features

Close-ended questions such as age, gender, current degree program, work responsibilities, and marital status were adopted for the survey to classify the socio-demographic data of students who participated in the recent study.

#### Self-Reported Physical Symptoms

Self-reported physical symptom knowledge was gathered through a series of close-ended dichotomous scales to provide an understanding of the respondent’s experience of physical symptoms. A dichotomous scale (Yes or No) aimed to avoid respondents’ misunderstanding and misinterpretation when reacting to their physical symptoms was used. Following consultation with medical professionals who had experience dealing with COVID-19 infected patients; a list of psychological symptoms was selected in addition to closeness toward COVID-19 symptoms. Concerning the experiences of COVID-19 symptoms, respondents were asked to reflect on their self-assessment or personal interpretation.

#### Home-Quarantine Activities and Particular Stressors of COVID-19

Home-quarantine activities and social stressors associated with COVID-19 are viewed as impacting psychological distress and poor mental health. The current study highlights students’ psychological well-being and mental health effects due to the lockdown situation. Among these variables, quarantine practices have been subjected to social and natural routine disturbances that negatively trigger students’ psychological well-being. There were options such as online social networking, recreational activity (e.g., entertainment show), physical exercise, educational activity, and scope for quarantine activity checklist to mention all other activities except those mentioned above. The scale was adopted from the study of Lima et al. (2020); four-point Likert scales ranging from 0 = not at all to 3 = sometimes in this 12 items-based scale. In addition to these practices, particular COVID-19 stressors, such as financial instability, fear of infection, and others were coordinated based on emerging literature on COVID-19 and students’ mental health (Khan et al., 2020).

#### Stress, Anxiety, and Depression (Psychological Well-Being and Mental Health)

*The student’s psychological well-being and mental health* were measured by assessing the self-reported level of stress, anxiety, and depression scale 21 from the Lovibond and Lovibond (1995) research. This scale comprises 21 questions that were split evenly between stress, anxiety, and depression. A four-point Likert scale ranging from 0 = never a problem, 1 often a problem, 2 = always a problem, to 3 = often a problem follows this scale. Examples include “I found it difficult to wind down” (stress), “I was aware of my mouth’s dryness” (anxiety), and “I did not seem to experience any good feeling at all” (depression; Alim et al., 2014). Examples include “I found it difficult to wind down” (stress).

#### Event-Specific Distress

The concept behind the adoption of event-specific distress was to evaluate student’s experience of any particular incident relevant to stress (Horowitz et al., 1979). The frequency of four-point Likert scales ranged from 0 = not at all to 3 = sometimes in this 15 items-based scale. Examples include “I had trouble falling asleep or staying asleep, due to images or feelings that came into my mind” (intrusion) and “I kept away from reminders of it” (avoidance). This scale was previously used during the COVID-19 outbreak in China (Li et al., 2020b; Zhang and Ma, 2020) and Bangladesh (Khan et al., 2020).

### Research Analysis

Perceptions linked to COVID-19 physical symptoms, home quarantine behaviors, COVID-19 particular stressors, and students’ psychological and mental well-being hierarchical regression modeling (HLM) were introduced to explore the proposed connection between sociodemographic variables. To satisfy the presumption of a direct relationship, the purpose of assessing these relationships was to ensure the existence of association and the essence of association between variables. According to Rutter and Gatsonis (2001), “the importance of this method is influenced by concentrating only on deviation from the mean, removing possible confounds that might arise from the rating patterns of individuals.” The final analysis is provided in various models that helped compare and analyze the intensity of different accessible models.

## Findings and Results

Table 1 reports a statistical analysis between them to summarize the correlation between sociodemographic variables and the students’ psychological stability and mental health. According to Table 1, with unmarried status (78.7%), most of the respondents lied between the brackets of 20 and 29 years (49.2%). Among these students, their Master’s degree was mainly checked (39.1%), and few of them had work responsibilities (27.6%). The proportion of male and female involvement was not very different from that of each other. There was a greater correlation between the age of students with perceived depression level (*B* = 0.19, *SE* = 0.01: *p* < 0.05) and event-specific discomfort (*B* = 0.23, *SE* = 0.04: *p* < 0.05) than stress and anxiety. On the other hand, after stress level (*B* = 0.26, *SE* = 0.04: *p* < 0.05), the degree of complexity and sophistication in the current level of respondent was found to have a strong effect on depression (*B* = 0.28, *SE* = 0.02: *p* < 0.01). The combined effect of marital obligations and job duties has less effect on the students’ mental health, which may be attributed to the lower number of individuals identified as married and having job duties. The proportion of variance by sociodemographic factors is high in the degree of depression relative to all these models. It highlights the strong effect of depression on students’ mental health, affecting stress or anxiety over longer periods.

**Table 1:** Connexion between sociodemographic features and psychological well-being and mental health of students.

N %		Model 1 Stress		Model 2 Anxiety		Model 3 Depression		Model 4 Event specific distress	
Indicators		*B*	*SE*	*B*	*SE*	*B*	*SE*	*B*	*SE*
**Age**									
30–39	27%	0.06	0.03	0.13	0.08	0.19^*^	0.01	0.23^*^	0.04
20–29	49.2%								
>20	23.8%								
**Gender**									
Male	42.7%	0.23^*^	0.02	0.19	0.03	0.18	0.08	0.19^*^	0.01
Female	57.2%								
**Current degree**									
Ph.D. Scholars	21.8%	0.26^*^	0.04	0.21^*^	0.07	0.28^**^	0.02	0.17	0.05
MPhil	19.4%								
Masters	39.1%								
Graduates	20.7%								
**Marital status**									
Married	21.2%	0.11	0.02	0.09	0.05	0.16	0.08	0.05	0.07
Unmarried	78.7%								
**Job duties**									
Yes	27.6%	0.16	0.05	0.13	0.08	0.07	0.03	0.08	0.04
No	72.4%								
*R*^2^		0.18		0.14		0.21	0.17		
*F*		44.97		23.79		41.43	21.89		

*R*^2^, *R* square; *B*, Beta value; *F*, *F* statistics.

* *p* < 0.05;

** *p* < 0.01.

Students’ experience linked to the unique physical symptoms of COVID-19 on their psychological well-being and mental health during quarantine are recorded in Table 2. As per the statistical study, when they encountered high fever, most of the respondents were affected and observed the same COVID-19 positive symptoms (*B* = 0.31, *SE* = 0.04: *p* < 0.01). In normal circumstances, high fever has a negative effect on the students’ psychological well-being, but the degree of impact was not high according to the standards. Stress (*B* = 0.24, *SE* = 0.01: *p* < 0.05) and ESD (*B* = 0.19, *SE* = 0.08: *p* < 0.05) were predicted for the incidence of cough or sore throat in students. The explanation for stress is that the temporary stress of COVID-19 symptoms can be expected. One of the major symptoms of exhaustion impacts the students’ psychological well-being and mental health, but the severity of the effect is not significant. However, stress (*B* = 0.32, *SE* = 0.05: *p* < 0.01), anxiety (*B* = 0.26, *SE* = 0.07: *p* < 0.05), depression (*B* = 0.21, *SE* = 0.09: *p* < 0.05), and ESD (*B* = 0.22, *SE* = 0.09: *p* < 0.05) were the worst affected by breathing difficulties during COVID-19. Similarly, stress levels (*B* = 0.29, *SE* = 0.07: *p* < 0.05), anxiety (*B* = 0.23, *SE* = 0.09: *p* < 0.05), and depression (*B* = 0.32, *SE* = 0.08: *p* < 0.01) were positively influenced by the influence of lost taste and smell. The cumulative effect of the physical symptoms of COVID-19 influences the psychological well-being and mental health of the student. However, the proportion of variance in the experience of COVID-19 physical symptoms across all four models is high in Model 4 (see Table 2). It explains that the level of distress increases when students come in contact with COVID-19 news, social media conversations, or information exposure. It can be concluded that having physical symptoms associated with COVID-19 perception can cause anxious thoughts. The effect will only be for shorter periods when a case is encountered.

**Table 2:** Connexion between the presence of psychical symptoms and psychological well-being and mental health of students.

Indicators	Model 1 Stress		Model 2 Anxiety		Model 3 Depression		Model 4 Event specific distress	
	*B*	*SE*	*B*	*SE*	*B*	*SE*	*B*	*SE*
High fever	0.11	0.04	0.07	0.08	0.09	0.05	0.31^**^	0.04
Cough/Sore throat	0.24^*^	0.01	0.19	0.03	0.17	0.06	0.19^*^	0.08
Fatigue	0.07	0.03	0.04	0.06	0.11	0.05	0.02	0.07
Breathing issues	0.32^**^	0.05	0.26^*^	0.07	0.21^*^	0.09	0.22^*^	0.09
Loss of taste or smell	0.29^*^	0.07	0.23^*^	0.09	0.32^**^	0.08	0.15	0.04
*R*^2^	0.23		0.16		0.11		0.34	
*F*	32.54		54.21		43.72		25.11	

*R*^2^, *R* square; *B*, Beta value; *F*, F statistics.

* *p* < 0.05;

** *p* < 0.01.

The association between home quarantine operations and students’ mental health during isolation is shown in Table 3. The negative correlation between social networking and depression (*B* = −0.29, *SE* = 0.04: *p* < 0.01) was shown to clarify the internet’s positive position during the quarantine period. However, according to COVID-19 figures, photos, and death tolls, it also positively impacted on event-specific anxiety (*B* = 0.23, *SE* = 0.01: *p* < 0.01). Educational events during the quarantine process played an important role in decreasing subscale mental health steps. On the other hand, negative aspects of increasing event-specific anxiety were also caused (*B* = 0.19, *SE* = 0.08: *p* < 0.05). The level of stress (*B* = −0.21, *SE* = 0.04: *p* < 0.05) and depression (*B* = −0.28, *SE* = 0.02: *p* < 0.01) was consistently decreased by the physical activities of students. Similarly, stress reduction (*B* = −0.27, *SE* = 0.02: *p* < 0.05) and anxiety (*B* = −0.25, *SE* = 0.03: *p* < 0.05) were highly affected by leisure behaviors during quarantine. The proportion of the difference between home quarantine behaviors in the event of stress is high compared to all these models. In terms of stressful feelings, it highlights the strong effect on students’ mental health, which appears to impair students’ thinking habits, focus, and ability to cope.

**Table 3:** Connexion between home quarantine activities and psychological well-being and mental health of students.

	Model 1 Stress		Model 2 Anxiety		Model 3 Depression		Model 4 Event specific distress	
	*B*	*SE*	*B*	*SE*	*B*	*SE*	*B*	*SE*
Social networking	−0.06	0.03	−0.03	0.07	−0.29^**^	0.04	0.23^**^	0.01
Educational activities	−0.13	0.02	−0.19	0.03	−0.08	0.09	0.19^*^	0.08
Psychical activities	−0.21^*^	0.04	−0.17	0.04	−0.28^**^	0.02	−0.08	0.04
Recreational activities	−0.27^*^	0.02	−0.25^*^	0.03	−0.16	0.03	−0.05	0.03
*R*^2^	0.25		0.14		0.18		0.19	
*F*	23.34		34.41		21.72		32.11	

*R*^2^, R square; B, Beta value; F, *F* statistics.

* *p* < 0.05;

** *p* < 0.01.

Situational variables are often involved in persuading bad psychological and mental well-being apart from personal qualities and characteristics. The relation between COVID-19 unique stressors and students’ mental health during isolation is shown in Table 4. The linear relationship between insufficient COVID-19 knowledge and the negative impact prediction of event-specific distress (*B* = 0.23, *SE* = 0.04: *p* < 0.01). It describes that students feel overwhelmed during quarantine when they experience a lack of knowledge directly related to COVID-19. Fear of being infected significantly increases the symptoms of depression (*B* = 0.18, *SE* = 0.02: *p* < 0.05). The occurrence of signs of depression is primarily due to the persistent rise in patients. The highest negative effect on stress (*B* = 0.31, *SE* = 0.04: *p* < 0.01), anxiety (*B* = 0.21, *SE* = 0.01: *p* < 0.05), depression (*B* = 0.38, *SE* = 0.03: *p* < 0.01), and ESD (*B* = 0.37, *SE* = 0.01: *p* < 0.01) was predicted by financial instability of all these COVID-19 unique stressors. It illustrates the financial problems, and the present situation is being robbed of by work market situations. Media attention also intervenes in this relationship, but the degree of impact is not important in either model. The proportion of variance of COVID-19 unique stressors in the case of stress is high compared to all these models. In terms of stressful thinking, this illustrates the strong effect on students’ psychological well-being and mental health.

**Table 4:** Connexion between COVID-19 specific social stressors and psychological well-being and mental health of students.

	Model 1 Stress		Model 2 Anxiety		Model 3 Depression		Model 4 Event specific stress	
	*B*	*SE*	*B*	*SE*	*B*	*SE*	*B*	*SE*
Inadequate information	0.16	0.03	0.13	0.03	0.11	0.05	0.23^**^	0.04
Fear of infection	0.23	0.02	0.27	0.03	0.18^*^	0.02	0.09	0.07
Financial uncertainty	0.31^**^	0.04	0.21^*^	0.01	0.38^**^	0.03	0.37^**^	0.01
Future uncertainty	0.23^*^	0.02	0.29	0.07	0.26^*^	0.09	0.15	0.03
Inadequate food supply	0.11	0.07	0.04	0.01	0.03	0.05	0.03	0.04
Media exposure	0.13	0.06	0.19^*^	0.03	0.23^*^	0.03	0.16	0.06
*R*^2^	0.31		0.21		0.23		0.26	
*F*	34.62		34.71		27.61		21.89	

*R*^2^, *R* square; *B*, Beta value; *F*, *F* statistics.

* *p* < 0.05;

** *p* < 0.01.

## Discussion

Like other people, students have faced complex natural challenges in the face of the current pandemic situation (Islam and Siddika, 2020). In this list of challenges, the main factors are health insecurity, job unpredictability, family aspirations, affected coping abilities, and physical challenges (Roy et al., 2020). This pandemic condition does not impact students’ physical capabilities but has also placed a more significant strain on mental health and psychological well-being (Wang et al., 2020). In compliance with the statistical analysis obtained from the data collected after being quarantined by university students. In addition, because of quarantine and self isolation many of students reported that pre-existing mental health issues are getting worst (Sahu, 2020).

University/college students in this stage of COVID-19 are more vulnerable to mental health problems. The current study results illustrate the process of change that underlines the detrimental effects on the students’ psychological well-being and mental health. The challenge of focusing on online classes was expressed by many of the students in line with results of Sahu (2020), which further triggered anxiety and guilt. A majority of students suffer from stress according to the subscales of psychological and mental well-being. In some situations, it turns to depression (Anjum et al., 2019). Among all other variables, the frequency of event-specific distress (ESD) is greater, which indicates that when they specifically experience any COVID-19 data, students feel more mental discomfort (Rubin and Wessely, 2020). Several respondents have reported extreme feelings of seclusion, helplessness, and panic attacks, leading to changing sleeping patterns and eating habits. Changes in mood swings and lifestyles may have a positive effect on the manifestation of symptoms of depression, according to Duan and Zhu (2020). Authentic leadership has a significant role in employee creativity (Sarfraz et al., 2019a).

The literature that has concentrated primarily on students’ mental health is scarce so far (Son et al., 2020). However, this initiative records the immediate concerns regarding mental health problems and students’ recommendations to deal with them (Cao et al., 2020). Therefore, rather than only concentrating on academic success, academic institutions need to establish countermeasures to preserve their students’ psychological health. The institutional capacity to work is not limited to the effectiveness of their technical skills but to how they deal with stressful and scary circumstances (Liu et al., 2020). Building the skills to cope with the current pandemic situation will require a considerable amount of formal research, practical education, and interventions (Nelson et al., 2020). In view of the current results, there is an emerging need to raise awareness of students’ mental health concerns (Duan and Zhu, 2020). The education support structure, career counseling, and non-conventional educational parameters of the higher education system need to be maintained immediately.

Nonetheless, some limitations should be overcome in future studies. Firstly, the sample size of current research is limited relative to the perspective horizon. Generally, studies with such schema have a large sample size to overcome the biases. To create a general understanding and compatible coping strategies, the sample size should increase in future studies with a similar understanding and population (Zhai and Du, 2020a). Second, the cross-sectional methodology design was mainly implemented during COVID-19 and quarantine to collect responses due to time constraints. This information gathering approach can only establish a short-term and incomplete view of students’ mental health challenges. Future researches can consider longitudinal design to check the psychological health and similar conditions. Third, but without any requirements or conditions, data was gathered from students. However, this analysis follows the methodology of snowball sampling, which can create constraints and generalizability issues. Future research is necessary to rely on probability sampling methods to prevent unjustified results from restricted disciplines. Finally, to build an in-depth understanding of poor psychological well-being and mental health, the latest study focuses on exploring quantitative analysis rather than a more mixed approach (Wang et al., 2020). Further research is needed to determine the pandemic’s impact on the mental well-being and well-being of students in its later stages after the peak period (Xie et al., 2020).

## Conclusion

An exceptional disturbance has been generated by the persistent realities of COVID-19 and related stressors and expanded altered behaviors. Tertiary education institutions have switched to an emergency online learning format due to physical distancing steps introduced in response to COVID-19, which will be expected to intensify academic stressors further. This research is currently offering assistance to mitigate the overall effects of COVID-19 on higher education students’ mental health. Evidence from the current study has supported the need to mitigate the adverse effects of COVID-19 and improve economic sustainability measures. Emerging studies examining the effects of COVID-19 on mental well-being have established an increased prevalence of moderate-to-severe self-reported depressive and anxious symptoms among students that indicate the widespread effects of confusion and health-related fears.

## Data Availability Statement

The raw data supporting the conclusions of this article will be made available by the authors, without undue reservation.

## Ethics Statement

The studies involving human participants were reviewed and approved by Ethics Review Committee from “COMSATS University Islamabad.” Written informed consent for participation was not required for this study in accordance with the national legislation and the institutional requirements.

## Author Contributions

HL conceptualized and supervised the whole study. HH contributed in the design, write-up, data collection, and analysis. MZ aided in the write-up and data collection. All authors contributed in reading and approving the final version of manuscript.

### Conflict of Interest

The authors declare that the research was conducted in the absence of any commercial or financial relationships that could be construed as a potential conflict of interest.
